# Human cues in eHealth to promote lifestyle change: An experimental field study to examine adherence to self-help interventions

**DOI:** 10.1016/j.invent.2024.100726

**Published:** 2024-02-08

**Authors:** Talia R. Cohen Rodrigues, David R. de Buisonjé, Thomas Reijnders, Prabhakaran Santhanam, Tobias Kowatsch, Linda D. Breeman, Veronica R. Janssen, Roderik A. Kraaijenhagen, Douwe E. Atsma, Andrea W.M. Evers, the BENEFIT consortium

**Affiliations:** a Health, Medical, and Neuropsychology Unit, Leiden University, the Netherlands; b Centre for Digital Health Interventions, Department of Management, Technology, and Economics, ETH Zurich, Zurich, Switzerland; c Instiute for Implementation Science in Health Care, University of Zurich, Zurich, Switzerland; d School of Medicine, University of St.Gallen, St. Gallen, Switzerland; e Department of Cardiology, Leiden University Medical Center, the Netherlands; f NDDO Institute for Prevention and Early Diagnostics (NIPED), Amsterdam, the Netherlands; g Vital10, Amsterdam, the Netherlands; h Department of Psychiatry, Leiden University Medical Center, Leiden, the Netherlands; i Medical Delta, Leiden University, Technical University of Delft, Erasmus University Rotterdam, the Netherlands

**Keywords:** eHealth, Digital health, Lifestyle change, Physical activity, Intervention, Conversational agent, Chatbot, Adherence, Working alliance

## Abstract

eHealth lifestyle interventions without human support (self-help interventions) are generally less effective, as they suffer from lower adherence levels. To solve this, we investigated whether (1) using a text-based conversational agent (TCA) and applying human cues contribute to a working alliance with the TCA, and whether (2) adding human cues and establishing a positive working alliance increase intervention adherence. Participants (*N* = 121) followed a TCA-supported app-based physical activity intervention. We manipulated two types of human cues: visual (ie, message appearance) and relational (ie, message content). We employed a 2 (visual cues: yes, no) x 2 (relational cues: yes, no) between-subjects design, resulting in four experimental groups: (1) visual and relational cues, (2) visual cues only, (3) relational cues only, or (4) no human cues. We measured the working alliance with the Working Alliance Inventory Short Revised form and intervention adherence as the number of days participants responded to the TCA's messages. Contrary to expectations, the working alliance was unaffected by using human cues. Working alliance was positively related to adherence (*t*(78) = 3.606, *p* = .001). Furthermore, groups who received visual cues showed lower adherence levels compared to those who received relational cues only or no cues (*U* = 1140.5, *z* = −3.520, *p* < .001). We replicated the finding that establishing a working alliance contributes to intervention adherence, independently of the use of human cues in a TCA. However, we were unable to show that adding human cues impacted the working alliance and increased adherence. The results indicate that adding visual cues to a TCA may even negatively affect adherence, possibly because it may create confusion concerning the true nature of the coach, which may prompt unrealistic expectations.

## Highlights

• Effects of human cues are rarely studied in text-based conversational agents (CA).
• Incorporating human (relational and visual) cues did not affect the working alliance.
• Yet, a good established working alliance contributed to higher adherence levels.
• Adding visual cues decreased adherence, possibly due to confusion about the CA's true nature.

## 1 Introduction

A healthy lifestyle has a positive effect on the number of disease-free years in an adult's life (Nyberg et al., 2020). A multicohort study showed that meeting the recommended physical activity levels, BMI, smoking behavior, and alcohol consumption would lead to an increase of 9.9 disease-free years for men and 9.4 of disease-free years for women (Nyberg et al., 2020). Lifestyle interventions are therefore widely recommended to improve health outcomes such as blood pressure or cholesterol levels (Piepoli et al., 2016). Long-term maintenance of recommended lifestyle behaviors is difficult for most people, yet the uptake and maintenance of lifestyle behaviors can be facilitated by the use of eHealth, which can be defined as the use of new information and communication technology, especially internet technology, to support or enhance health and health care (Barak et al., 2009). An increasing amount of eHealth lifestyle interventions are being developed (Thomas and Bond, 2014), which are shown to be effective in improving lifestyle behaviors (eg, physical activity) and consequently reduce risk factors that are associated with lifestyle-related diseases (eg, high blood pressure, high cholesterol) (Beishuizen et al., 2016; Lunde et al., 2018). Within eHealth interventions, support can either be provided by a human professional (human-supported interventions), or automatically through computer technology, meaning that there is no human guidance or human professional involved. Interventions in which there is no support offered through human contact, but only automated support by computer technology, are defined as self-help interventions (Barak et al., 2009). For this reason, self-help interventions are generally easier and cheaper to widely implement to a larger and more varied audience as they require no involvement from healthcare professionals, who may lack time or insufficient experience to additionally offer lifestyle support (Brotons et al., 2005; Jallinoja et al., 2007; Jansink et al., 2010).

There is however a downside to self-help interventions. Adherence, or the extent to which a person uses the eHealth intervention as intended, is often problematic (Kelders et al., 2012; Kelders et al., 2011; Murray et al., 2013; Wangberg et al., 2008). This means that people use self-help interventions less frequently or stop using it earlier than necessary for the intervention to be optimally effective. However, this does not imply that support of a healthcare professional is always necessary for optimal results. Meta-analyses revealed that human contact with a nonprofessional is enough to both ensure intervention effectiveness and prevent individuals from dropping out of the intervention (Etzelmueller et al., 2020; Karyotaki et al., 2018; Richards and Richardson, 2012; Smith et al., 2012). It seems that the mere involvement of another human being, or something that is perceived as having human traits (Haslam et al., 2008), rather than professional guidance is the key ingredient within human-supported interventions. The underlying reason for the found effects of human contact within interventions could be the participants' need for a personal relationship with a care provider (Brandt et al., 2018). In clinical practice this relationship is called the working alliance, which is defined as the degree to which a healthcare professional and patient are involved in a useful and collaborative working relationship (Hatcher and Barends, 2006). Although the concept of working alliance originated within psychotherapy (Bordin, 1979), it has more recently been applied to the domain of lifestyle interventions (Goldberg et al., 2013; Hauser-Ulrich et al., 2020; Kowatsch et al., 2021a). The quality of the working alliance depends on several factors such as the level of agreement on treatment goals, on tasks that must be performed to reach treatment goals, and on the quality of the relationship between healthcare professional and patient (Bordin, 1979; Horvath and Greenberg, 1989). The establishment of a good working alliance promotes intervention adherence and effectiveness, both in face-to-face interventions (Goldberg et al., 2013; Martin et al., 2000) as well as in eHealth interventions with human contact (Flückiger et al., 2018; Sucala et al., 2012). In addition, individuals are also able to form a working alliance with computers (Nass and Moon, 2000; Reeves and Nass, 1996). Individuals can interact with computers as they would do with human beings and apply similar social rules and heuristics. For example, people tend to communicate with their smartphone (eg, Apple's Siri) in a similar way as they would do with another human being. The establishment of a working alliance in eHealth interventions can also lead to more positive treatment outcomes (Hauser-Ulrich et al., 2020; Kowatsch et al., 2021a; Bickmore et al., 2010; Clarke et al., 2016; Kowatsch et al., 2021b).

So, how can we establish a working alliance in self-help interventions without human contact? For this, so-called conversational agent (CA) can be employed. CAs can be defined as computer-based agents which can mimic human-like conversational behavior such as responding to input, generate output, apply turn-taking) (Cassell et al., 1999). With these characteristics, they can provide automated support in eHealth interventions (eg, home exercising (Kowatsch et al., 2021b)) to promote adherence to lifestyle behaviors. An embodied conversational agent (ECA) is visually present on screen and can provide non-verbal cues (eg, hand gestures), while a text-based conversational agent (TCA) is able to communicate with text only (Kowatsch et al., 2018). A TCA has the advantage of being easier to develop, being easier to apply in a mobile app, and is therefore more suitable for widespread implementation (Kowatsch et al., 2018). Studies demonstrate that people show more relational behaviors, such as facial expressions, and are more positive about the interaction when they believe that their interaction partner is a human being rather than a computer technology (Aharoni and Fridlund, 2007; Appel et al., 2012). To enhance these perceptions while interacting with CAs, human cues could be applied, such as an avatar of a human being, a human tone-of-voice (Sah and Peng, 2015), or lower speed of feedback (Kelders et al., 2015). Furthermore, human cues in textual communication could be mimicked by adding emoticons (Walther and D'addario, 2001). Besides the appearance of the messages, human cues could also be applied to the content of its messages. Conversation rules which are often used by humans to established a relationship, such as humor, empathy and small talk could also be incorporated as human cues in CA (Bickmore et al., 2005; Schulman and Bickmore, 2009). Studies with CAs show that applying such human cues to the interaction increases the working alliance users experience with the CA (Bickmore et al., 2005) and their intention to use the CA (Lisetti et al., 2013).

To conclude, although self-help intervention studies with TCAs have been conducted before to examine their effect on psychological outcomes, only a small number of them focused on improving lifestyle behaviors (Tudor Car et al., 2020). Furthermore, the effects of human cues are predominantly tested with ECAs (eg (Bickmore et al., 2005; Lisetti et al., 2013)). Therefore, little is known about how human cues affect the working alliance when applied in TCAs, or how the working alliance affects adherence to TCA-supported interventions. Furthermore, the majority of studies tested the effects of either using human cues or not (eg (Sah and Peng, 2015; Kelders et al., 2015; Bickmore et al., 2005; Schulman and Bickmore, 2009; Lisetti et al., 2013)), while it is more interesting to test the effect of different types of human cues and possible interaction effects when combining human cues.

### 1.1 The present study

In this study, we will examine the impact of human cues in TCA on establishing a working alliance in a self-help lifestyle intervention. In addition, we will examine the impact of human cues and the working alliance on participant's intervention adherence. With regard to human cues, we will focus on both visual cues (ie, appearance of the message and TCA) and relational cues (ie, content of the message). We will test the following hypotheses. First, human cues will improve the working alliance people experience with TCA. Second, an established working alliance and application of human cues will promote participants' adherence to the lifestyle intervention. Finally, the working alliance will mediate the effect of human cues on adherence. To test our hypotheses, we developed a self-help intervention mobile application with which the participant could interact with a TCA and in which we manipulated both visual and relational cues.

## 2 Material and methods

### 2.1 Study design and procedure

The three-week field experiment was conducted in March and April 2020. To test our hypotheses, we employed a 2 (visual cues: yes, no) × 2 (relational cues: yes, no) between-subjects design, resulting in four experimental groups: (1) visual and relational cues, (2) visual cues only, (3) relational cues only, or (4) no human cues. Power calculations (G*Power) (Faul et al., 2007) identified that we needed a minimum sample size of 128 to detect a medium between-group effect (f = 0.25) of cue-type with an alpha of 0.05 (ANOVA with 4 groups). Given the high attrition rates in similar studies (eg, (Hauser-Ulrich et al., 2020; Kramer et al., 2019)), we aimed to recruit about double the required number of participants (ie, 256).

We recruited healthy participants using voluntary response sampling with flyers on the university campus and via social media (eg, personal social media channels of thesis students involved in the project, public student social media groups). Inclusion criteria were that participants should be aged between 18 and 30 years old, were able to work on their level of physical activity (ie, based on a negative response to all questions of the Physical Activity Readiness Questionnaire (PAR-Q) (Thomas et al., 1992)), willing to work on their level of physical activity, have access to a smartphone running iOS or Android, and would have sufficient proficiency in English. Participants were promised that after completion of the experiment, they would enroll in a lottery with the chance of winning one of three Fitbit devices, or one of 100 webshop vouchers worth €10,-. In addition, first-year students could receive credits required to complete their first bachelor year for their participation.

After recruitment, participants joined a waitlist until the start of the screening and onboarding on Monday March 16th 2020. Participants had to wait a maximum of three weeks before the start of the experiment. A week before the start of the experiment, participants were asked to provide digital informed consent and fill in the screening survey assessing the inclusion and exclusion criteria. Immediately after providing their consent and being screened as eligible to participate, participants received a link to the iOS or Android app store to download the Benefit StepCoach app. Once the app was downloaded, participants were asked to go through the onboarding procedure to correctly configure the app (eg, allowing push messages and access to step count data via Apple Health or Google Fit), and to complete the baseline survey. Participants were reminded through emails and text messages to complete the onboarding and baseline survey (measuring demographics and baseline characteristics) after 3, 4 and 5 days. Participants were excluded if they did not finish onboarding before the start of the experiment. An automated mechanism within the app allocated participants to one of the four conditions. All participants would start simultaneously in the three-week (21 days) experiment on Monday March 23rd 2020. A 3-week intervention duration was chosen as this would be enough time to establish a working alliance with the TCA (given that a relationship can be established after a single interaction, eg (Bickmore et al., 2010; Schulman and Bickmore, 2009)), and to be able to measure changes in adherence to the intervention. Each day, the TCA would send the participants one or several short exercises to complete that day (eg, quiz or worksheet, see Appendix 1 for an overview of daily exercises) via a push notification. After completing the final survey on day 22 (measuring working alliance), participants would receive the debriefing. The study was approved by the Psychology Research Ethics Committee of Leiden University (2020-02-06-T. Reijnders-V2-2056), and the analyses were preregistered via the Center for Open Science (Cohen Rodrigues and Reijnders, 2020).

### 2.2 Participants

In total, 269 participants were recruited during the wait list period, and were invited to the screening survey. Of these, 43 participants did not meet the inclusion criteria. Of the remaining 226 eligible participants, 127 participants downloaded the app, after which 121 participants completed the baseline measurement (attrition rate of 45 %). We were unable identify reasons for dropping out between the recruitment and baseline measurement.

### 2.3 Benefit StepCoach intervention

The aim of the intervention was to enhance participants' physical activity levels by increasing daily step counts. The intervention was based on a combination of important behavior change techniques (BCTs) (Michie et al., 2013), such as providing participants with information on health consequences, setting and reviewing of health behavior goals, and providing social rewards such as appraisal of the participant's efforts. These are intervention components designed to regulate behavior (such as physical activity) by reinforcing factors that facilitate behavior change, and mitigating factors that hinder behavior change (Michie et al., 2013). Participants would receive daily exercises based on BCTs, which would take about 5 to 10 min each day to complete (see Appendix 1 for an overview of all daily exercises). The Transtheoretical Model of health behavior change (Prochaska and Velicer, 1997) was used to develop specific exercises that match each phase of the model, as research shows that choosing exercises that fit within the pre-contemplation, contemplation, preparation, action, and maintenance stage stimulates user adherence and effective behavior change. Furthermore, the model would be applicable to our intervention as it has been previously used to target a wide range of health behaviors, including physical activity (Prochaska et al., 1994). For example, in the pre-contemplation phase we let participants formulate why they would like to improve their physical activity, and in the contemplation phase we let participants formulate pros and cons of behavior change. Later, in the preparation phase, we asked participants to formulate a concrete step goal. In the action phase, participants received action-planning and problem-solving exercises to help them reach their goal. Finally, in the maintenance phase, participants reviewed their previous successes to help them maintain the new behavior in the future. For an overview of all the exercises (ie, active ingredients of the intervention) per phase of the Transtheoretical model, see Appendix 1. The mobile application for our self-help intervention was developed with use of the open-source software of MobileCoach (www.mobile-coach.eu) (Filler et al., 2015; Kowatsch et al., 2017), which has been previously used for smartphone-based and chatbot-delivered behavioral interventions (eg, (Tinschert et al., 2019; Lee et al., 2011)). See Appendix 2 for more information about the technical implication. As we developed our own intervention, it was important to test whether it was actually effective in improving participants' physical activity levels. Therefore, we conducted some additional analyses, which showed us that the intervention significantly increased participants' step count independently of the experimental condition (see Appendix 3 for more details).

### 2.4 Text-based conversational agent

**Fig. 1:**
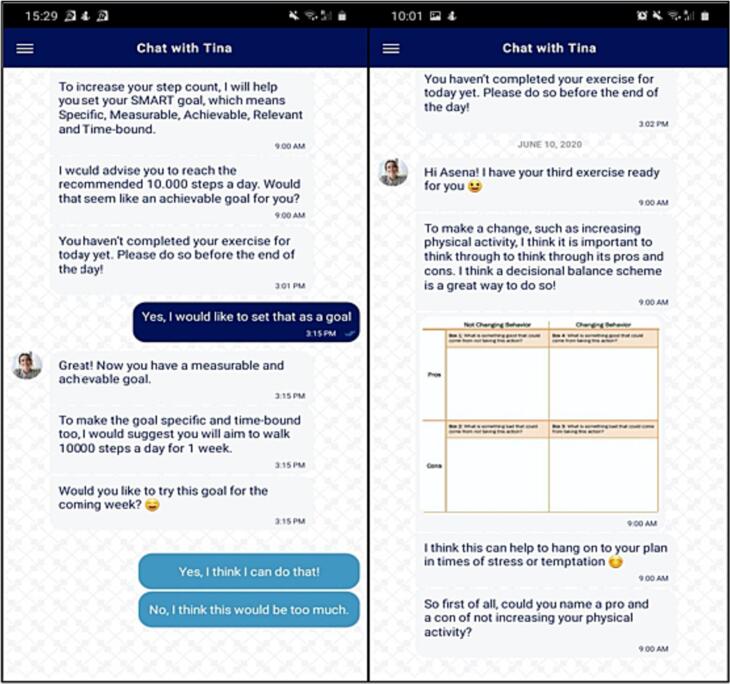
Screenshots of Benefit StepCoach app.

Participants interacted daily with a TCA, the virtual coach who delivered the intervention and offered various conversational turns. Via the chat feature, the TCA delivered daily exercises (see Appendix 1) and would respond to messages of the participants via conversational turns (see Fig. 1). All conversational turns were scripted. Each day would consist of two to four conversational turns. The first message would be sent in the morning at 9:00 am, and the following messages after a reply of the participant. If the participant would not reply on time, the TCA would send a reminder in the afternoon at 3:00 pm.

**Fig. 2:**
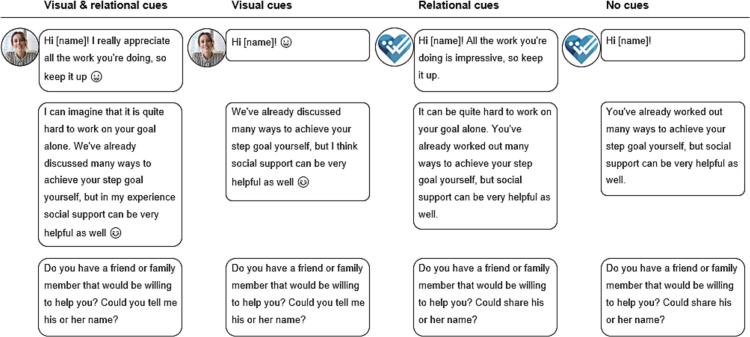
Example of conversational turns per condition.

Across the experimental groups, the exercises and feedback were identical, but the conversational turns differed in cue type the TCA used. We manipulated two types of human cues: (1) visual cues, which were related to the appearance of the message and the TCA (human avatar, use of emoticons, human tone-of-voice, and response delay), and (2) relational cues, which were related to the content of the messages, and to what extent these followed social scripts and human conversation rules (eg, showing empathy, self-disclosure, humor, small talk, and meta-relational communication) (see Fig. 2).

### 2.5 Measures

#### 2.5.1 Baseline measures

During the onboarding week before the start of the intervention, participants were asked to fill in several demographic questions on gender, age, nationality, and educational background. Furthermore, baseline level of physical activity was measured with the International Physical Activity Questionnaire Short Form (IPAQ-SF) (Craig et al., 2003). The questionnaire consists of seven items asking the participants about their time spent on vigorous and moderate physical activities, walking, and sitting during the previous week. The output is a MET (metabolic equivalent of task) score, representing the amount of energy used to carry out the reported physical activities. A higher score indicates a higher level of physical activity. The IPAQ-SF has been shown to have a high test-retest reliability, but minimal concurrent validity (Craig et al., 2003; Hatcher and Gillaspy, 2006). Therefore, baseline objective step count data was additionally retrieved from the participant's smartphone during the onboarding week.

#### 2.5.2 Working alliance

Participants' working alliance with the TCA was measured with a revised version of the Working Alliance Inventory Short Revised form (WAI-SR) (Hatcher and Gillaspy, 2006). The WAI-SR consists of 12 items to measure the experienced quality of the working relationship between patient and professional. All items were measured on a 5-point Likert-scale ranging from 1 (seldom) to 5 (always), subdivided in 3 subscales: agreement on tasks, agreement on goals, and bond. Questions were revised to fit the context of the study by using the words “coach”, “lifestyle” and “intervention” (eg, “The coach and I collaborate on setting lifestyle goals.”). A higher score indicates a better working alliance with the TCA. The WAI-SR has been shown to have sufficient test-retest reliability and criterion validity (Hatcher and Gillaspy, 2006), and our revised version showed to have a high internal consistency (Cronbach's α = 0.95).

#### 2.5.3 Adherence

Participants were marked as “adherent” for a particular day if they had replied to the final message of the TCA before the end of the day (12:00 pm at midnight). The final adherence measure was based on the number of days participants finished each daily session of conversational turns with the TCA. Given the 21 day duration of the intervention, the level of adherence over the whole study could range between 1 and 21 days, with higher number of days indicating a higher level of adherence.

### 2.6 Data analysis

All statistical analyses were conducted with SPSS (version 26; IBM Corp). We used pairwise exclusion to deal with missing data and a standard *P*-value of 0.05 was chosen to determine statistical significance. For the first hypothesis (human cues will improve the working alliance people experience with TCA), we performed a Kruskal-Wallis test, with working alliance as our outcome measure and cue condition as independent variable. We chose to conduct non-parametric tests given the small sample size of some groups (*N* < 25) and a non-normal distribution of our data. For the next hypothesis (working alliance and human cues will promote adherence to the intervention), we ran a regression analysis with working alliance as independent variable and adherence as outcome measure and performed a Kruskal-Wallis test with cue condition as independent variable and adherence as outcome measure. This was followed up by the analyses of specific post-hoc analyses to compare different cue groups in the form of Mann-Whitney *U* tests. For the final hypothesis (working alliance will mediate the effect of human cues on adherence) we planned to conduct a mediation analysis. However, the lack of significant differences in working alliances between groups made this analysis obsolete.

In our preregistration (Cohen Rodrigues and Reijnders, 2020) we also proposed to test intervention effectiveness. Our power calculations identified a minimum sample size of 128 to detect the expected effects of experimental groups on effectiveness. However, as we needed both a valid baseline step count and a minimum of 5 days of step count registered in the final week to calculate intervention effectiveness, we did not have enough power to run these analyses and detect this effect due to insufficient respondents. We therefore decided to report the analyses concerning intervention effectiveness only in Appendix 3.

## 3 Results

### 3.1 Demographics

A total of 121 participants completed the baseline measurement. These participants were on average 22.7 years (SD = 2.8) old, 84/121 (69 %) were female, 73/121 (60 %) were of Dutch nationality, and of 91/121 (75 %) their current or highest education level was bachelor's degree or higher. Comparative analyses of the demographic characteristics at baseline showed no significant differences between groups (see Table 1).

**Table 1:** Baseline demographic characteristics (*N* = 121).

Variable	Visual & relational cues (*n* = 31)	Visual cues (*n* = 24)	Relational cues (*n* = 29)	No cues (*n* = 37)	*P* value
Age in years					
Median (IQR^a^)	22 (4)	23 (3)	22 (3)	23 (4)	.968^d^
Mean (SD^b^)	22.65 (2.84)	22.71 (2.79)	22.76 (2.70)	22.54 (3.01)	
Gender, female, n (%)	26 (84)	12 (50)	21 (75)	25 (68)	.055^e^
Nationality, n (%)					.743^e^
Dutch	19 (61)	15 (63)	14 (48)	25 (67.5)	
German	3 (10)	3 (13)	6 (21)	5 (13.5)	
Other	9 (29)	6 (25)	9 (31)	7 (19)	
Education level, n (%)					.306^e^
High school	4 (13)	6 (25)	6 (21)	11 (30)	
Vocational school	1 (3)	1 (4)	0 (0)	1 (3)	
Bachelor's degree	17 (55)	14 (58)	21 (72)	18 (49)	
Master's degree or higher	9 (29)	3 (13)	2 (7)	7 (19)	
Physical activity level					
MET^c^ score (per week)					
Median (IQR^a^)	2552 (4150)	1506 (2986)	2268 (4730)	2477 (4331)	.134^d^
Mean (SD^b^)	4556 (5324)	2928 (5370)	3800 (3373)	3854 (3804)	
Average steps per day					
Median (IQR^a^)	2453 (2840)	1531 (2685)	3224 (3222)	2382 (2382)	.357^c^
Mean (SD^b^)	3282 (2289)	1912 (1557)	3266 (1601)	3361 (2616)	

a IQR = interquartile range.

b SD = standard deviation.

c MET = metabolic equivalent of task; ^d^Kruskal-Wallis test; ^e^Fisher's Exact test.

### 3.2 Working alliance

We found no significant difference in working alliance between the cue conditions, *H*(3) = 4.194, *p* = .24 (see Table 2 for median and IQR per group). However, we did find a positive relationship between working alliance and adherence, *β* = 0.378, *t*(78) = 3.606, *p* = .001, 95 % CI [0.108; 0.374]. These outcomes indicate that that adding human cues did not lead to a difference in working alliance with the TCA, but that participants who reported a better working alliance were more adherent to the intervention.

**Table 2:** Median and IQR^a^ per group of working alliance (measured after the final day of the intervention with the Working Alliance Inventory Short Revised form) and adherence (number of days participants finished the session of conversational turns).

Variable	Visual & relational cues	Visual cues	Relational cues	No cues	*P* value
Working alliance^a^					
Median (IQR^b^)	34 (18)	45 (18)	42 (8)	34 (13)	.24^c^
Adherence					
Median (IQR^b^)	6 (12)	7 (14)	16 (14)	14 (15)	.004^c^

a The N for working alliance (due to missing data): visual & relational cues: *n* = 19; visual cues: *n* = 14; relational cues: *n* = 22; no cues: *n* = 25.

b IQR = interquartile range.

c Kruskal-Wallis test.

### 3.3 Adherence

**Fig. 3:**
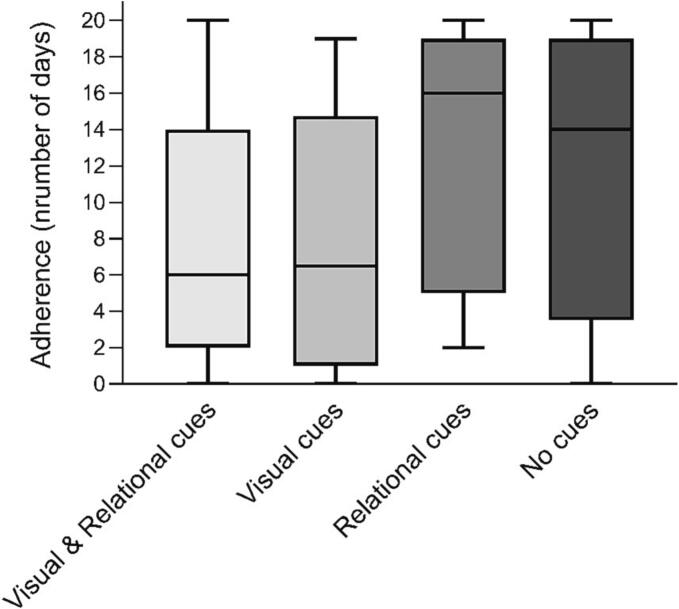
Boxplots of adherence (number of days participants finished the session of conversational turns) for the four experimental conditions.

We found a significant difference in adherence between the cue conditions, *H*(3) = 13.125, *p* = .004 (see Table 2 for median and IQR per group). By visually inspecting the medians, we saw that the differences between groups were not as expected (see Fig. 3). The contrast analyses showed that in the relational cues- and no cues-conditions there was a significantly higher adherence than in the other two conditions, *U* = 1140.5, *z* = −3.520, *p* < .001. However, adherence in the relational cues condition was not higher than in the no human cues condition *U* = 478.0, *z* = −0.760, *p* = .45. So contrary to what was expected, participants were less adherent to the intervention in the groups in which the TCA used visual cues compared to the groups without visual cues. Furthermore, when the TCA used relational cues, participants were not more adherent than when the TCA used no human cues at all.

## 4 Discussion

We investigated the impact of human cues in TCA on establishing a working alliance and in turn that impact on improving the adherence in a self-help lifestyle intervention. We found no differences in the effect of no, visual and/or relational human cues on establishing a higher quality working alliance with the TCA. Also, using visual or relational human cues did not lead to higher adherence. On the contrary, we found that the use of visual cues could even lead to lower adherence. However, we did find that a higher quality working alliance was related to a better adherence to the lifestyle intervention.

Our results did not show an effect of human cues on the reported working alliance with the CA. What is important to note is that many studies that did find this relationship concern ECAs (Bickmore et al., 2010; Bickmore et al., 2005), while we used a TCA. ECAs generally outperform text-based ones (Lisetti et al., 2013; Zalake et al., 2019), which can be explained by the additional range of design characteristics an ECA can make use of (Loveys et al., 2020). In one study though, there was no difference found between a TCA and an ECA, which the authors argued was due to the lack of incorporating non-verbal communication in the latter one (Friederichs et al., 2014). The inability of our (or any) TCA to use non-verbal communication, may be a reason that we did not find the effect of our TCA with human cues on working alliance as studies with ECAs did. Similar patterns occur in computer-mediated communication between humans, where people are limited in their use of non-verbal communication (Daft and Lengel, 1986). Text-based communication would not be rich enough to transfer ambiguous communication, such as communication aimed at relationship building (Daft and Lengel, 1986), and relationship building requires more time in text-based environments to reach the same quality as in face-to-face situations (Walther, 1992). This might also explain why we did not find an effect of using relational cues on adherence, a finding that contradicts previously mentioned studies with ECAs (Bickmore et al., 2005; Schulman and Bickmore, 2009). Moreover, in studies that did find an improved working alliance with a CA, either the interactions with the agent or in the intervention itself were longer compared to our study (Hauser-Ulrich et al., 2020; Bickmore et al., 2010). In other studies in which a high working alliance was reported within shorter periods of time, the interactions with the CA followed after introduction by a human healthcare professional (Kowatsch et al., 2021a; Kowatsch et al., 2021b). Therefore it seems likely that a TCA is less able to build a relationship with the user due to lack of non-verbal communication, however it could be that it requires either a longer time period or an introduction in a face-to-face setting to do so. So even though our findings do support that working alliance is an important mechanism within eHealth interventions, it remains unclear if and how it would be possible to foster a relationship with a TCA. Even though the development of an ECA requires more time and financial resources than a TCA, based on both our results and those of previous studies, we hypothesize that self-help eHealth interventions in practice would benefit more from incorporating an ECA. The difference between TCAs and ECAs and their applicability in successful eHealth interventions would be an important topic for future research.

We did find that people who reported a better working alliance with the CA were more adherent to the lifestyle intervention. This result is in line with studies about regular face-to-face interventions (Goldberg et al., 2013; Martin et al., 2000), digital therapy or treatment (Flückiger et al., 2018; Sucala et al., 2012), and self-help eHealth interventions (Hauser-Ulrich et al., 2020; Kowatsch et al., 2021a; Bickmore et al., 2010; Clarke et al., 2016; Kowatsch et al., 2021b). However, our results did not show the positive effects of visual elements that have been reported in previous studies (Bickmore et al., 2010; Sah and Peng, 2015; Bickmore et al., 2005; Schulman and Bickmore, 2009). Instead, we found that using visual cues led to a lower adherence to the intervention. We did not tell participants whether they would be coached by a human being or a computer. This lack of transparency, in combination with a human visual appearance, may have led to unrealistic high expectations that could not be met by the TCA and therefore frustration among users (Luger and Sellen, 2016). Although many studies show that not disclosing the nature of an automated chatbot has a positive effect on user perceptions (eg, perceived humanness of, or affinity with the chatbot) and user behavior (eg, being persuaded by the chatbot) (Hendriks et al., 2020; Shi et al., 2020; Skjuve et al., 2019), Mozafari and colleagues (Mozafari et al., 2020) show that the effects of disclosure depend on whether there are errors in the conversation with a chatbot. In their study with a customer-service bot, they found that when the chatbot was not able to solve a customer's issue, the customer's potential negative responses to these errors could be prevented by disclosing the chatbots true nature beforehand. Although our study concerned a lifestyle intervention rather than customer-service, similar mechanisms could be at play here. As visual cues might have caused participants to wrongly expect they were communicating with a human being and our CA was not always able to respond correctly to participant's messages (as the messages were preprogrammed), informing participants about the nature of the agent could have prevented unrealistic expectations and frustration. In addition, the type of avatar we used in the visual cues conditions might have played a role. We intentionally chose a younger- and healthy-looking female agent both because it resembles the psychology student population, and a young female peer agent is generally preferred in health coaching tasks (ter Stal et al., 2020a; Zhou et al., 2014). However, some literature suggests that male agents are preferred as athletic trainer, which might have influenced the results if our participants perceived the TCA to be an athletic coach rather than a health coach (ter Stal et al., 2020b). Furthermore, another study shows that non-ideal overweight agents are seen as more trustworthy and related to higher use intentions (van Vugt et al., 2009), which suggests our TCA might have been too slender and healthy looking for its task. All in all, future designers of eHealth interventions with TCAs could consider being transparent about the true nature of the CA, as it would make users more forgiving about possible imperfections of the automated feedback it provides. Furthermore, given the important influence of the type of visual cues, it would possibly be beneficial for future eHealth interventions to better match the visual cues of the TCA with the wishes of the user. For example, one could allow users themselves to choose the looks of the TCA that will support them. Future research could investigate whether such changes would improve adherence to self-help eHealth interventions.

### 4.1 Practical implications

Further knowledge about the development of CAs is not only relevant for researchers working in eHealth or human-computer science, but also for those involved in healthcare practice. eHealth is becoming increasingly relevant, which became especially evident during the COVID-19 pandemic (Bokolo, 2021). Therefore, it is necessary to develop eHealth tools that are efficient, and thus do not put further pressure on the workload of healthcare professionals, but at the same time fulfill the needs and wishes of patients. CAs would be suitable for developing self-help eHealth lifestyle interventions that do pay attention to the relationship with the user. Furthermore, our findings would not only be practically relevant for developing physical activity interventions, but eHealth lifestyle interventions in general. Therefore the findings of our study would be useful for developers that work on self-help eHealth lifestyle interventions, and indirectly for healthcare professionals who could help their patients in providing lifestyle support more easily.

### 4.2 Limitations and suggestions for future work

Besides the strengths of our study such as using a field experiment (with participants using an app-based intervention in real life), measuring objective behavioral data, and testing two different types of human cues, our study also had some limitations. In our preregistration, we proposed to also test intervention effectiveness, yet we did not have sufficient participants and thus power to do so. For reasons of transparency, we do report the analyses on intervention effectiveness in Appendix 3. It is also important to note that our sample size was generally on the small side and that we had problems with non-normality in our data. Although we used nonparametric tests to analyze our data, the results should be interpreted with caution. Even though we already recruited more participants than needed to account for possible dropouts, future studies may aim to recruit more than double the needed participants.

Furthermore, we did not inform our participants beforehand whether they were interacting with a computer or a human being. Therefore the expectations of people might have varied, which could have affected our results. Future studies could keep these expectations constant by being transparent about the true nature of the automated agent. Another option would be to manipulate the description of the CA to more closely represent a human being or a computer, and ask participants about their expectations towards support by a human being or computer, to additionally test expectation effects within self-help interventions.

Finally, to mimic human behavior, we intentionally chose to apply subtle human cues to our CA (eg, interweaving signs of empathy or small jokes into the feedback). However, participants might not have processed the messages of the agent elaboratively enough to notice these subtle cues, resulting in a lack of effects. Furthermore, because of this subtility, the different types of human cues might have differed too little between each other. We suggest that future studies investigate the differences in applying human cues in TCAs and ECAs. It would be interesting to know whether stronger cues are needed in TCAs to produce similar effects in ECAs, or whether longer interactions do lead to an improved working alliance, and thus adherence. Additionally, given our results, it would be interesting to investigate whether using non-verbal communication is indeed key to establishing a working alliance with a CA, and how to overcome the lack of non-verbal communication within TCAs.

### 4.3 Conclusions

In this study, we aimed to improve adherence to self-help eHealth lifestyle interventions by applying a TCA which uses (visual and relational) human cues. We replicated that creating a good working alliance with your coach improves adherence to lifestyle interventions. However, more future studies are needed to investigate whether and how factors that work for ECAs, in this case human cues, could also be applied to TCAs to further improve the working alliance and thereby adherence. Future studies could also investigate whether being transparent about the computer-based nature of a CA and thereby setting the right expectations would be important for success. Until future research provides us more insight, our findings suggest that self-help eHealth interventions in practice could possibly better invest in developing an ECA and be transparent about the true nature of the CA that is used. The knowledge gained from our and future studies could help us design better self-help interventions in the future creating higher levels of adherence, and in turn a healthier lifestyle for us all.

## CRediT authorship contribution statement

Study design (TRCR, TR); intervention and app design and development (TRCR, PS, TK, AK); data acquisition (TRCR, DRdB, AK); data analysis and interpretation (TRCR, TR, AWME); drafting the manuscript (TRCR, TR, PS, TK, AWME); manuscript revision (TRCR, TR, DRdB, PS, TK, LDB, VJ, RAK, DEA, AWME). All authors gave final approval and agree to be accountable for all aspects of the work ensuring integrity and accuracy.

## Declaration of competing interest

TK is affiliated with the Centre for Digital Health Interventions (CDHI), a joint initiative of the Institute for Implementation Science in Health Care, University of Zurich, the Department of Management, Technology, and Economics at ETH Zurich, and the Institute of Technology Management and School of Medicine at the University of St.Gallen. CDHI is funded in part by CSS, a Swiss health insurer. TK is also a cofounder of Pathmate Technologies, a university spin-off company that creates and delivers digital clinical pathways. However, neither CSS nor Pathmate Technologies was involved in this research. All other authors declare no conflicts of interest.

## Figures and Tables

**Table:** 

Day	TTM stage	BCTs	Exercise
1	Pre-contemplation	Goal setting	Formulate general goal *Participants are asked to describe what general health improvement they would like to achieve through increasing physical activity levels, and why this would be important to them in order to prepare their mindset for future behavior change.*
2		Information about health consequences	Quiz about behavior and health consequences *By doing the quiz, participants receive more knowledge about how (a lack of) physical activity would affect their health to help improve their attitude towards increasing their physical activity levels.*
3	Contemplation	Pros and cons	Decisional balance worksheet *Participants are asked to critically think about the pros and cons of changing and not changing their physical activity behavior to help them create recognition about advantages and disadvantages of engaging in higher levels of physical activity.*
4	Preparation	Goal setting	Formulate SMART goal *Participants are asked to create a specific, measurable, achievable, relevant and time-bound goal to help them start increasing their step count.*
5		Valued self-identity	Self-affirmation exercise *Participants are asked to think about values that are important to them and how physical activity fits with these values to help stick with the goal they have set.*
6		Prompts/Cues; Action planning	Formulate ‘If-then plan’ *Participants are asked to set a physical activity-related plan when a specific situation occurs to increase the chances of reaching their goal.*
7		Problem solving	Identify barriers and coping strategies *Participants are asked to think about potential barriers that might hinder reaching their physical activity goal and about solutions to overcome these to help them prepare for these situations.*
8		Self-monitoring	Implement short bursts of activities, and compare step-count to yesterday's *Participants are asked to think of a small activity for today that would increase their physical activity level, and to compare their results with yesterday to see how such small steps can help achieve their goal, and motivate them in applying these during the rest of the process.*
9		Action planning; Social support; Barrier identification	Plan physical activity challenge with other person; identify barriers and coping solutions *Participants are asked to involve a peer by asking them to join physical activity challenge to create social support in reaching their goal.*
10	Action	Instructions on how to perform health behavior	Quiz about performing physical activity *By doing the quiz, participants receive more knowledge about types of physical activity and how these increase their step count level to give them with new ideas to turn their intentions into action.*
11		Review behavioral goal(s)	Reflect on goals (day 4) and make adjustments (SMART) *Participants are asked to look back at their goal of day 4, and if needed, create a new specific, measurable, achievable, relevant and time-bound goal to help them increase their step count.*
12		Identification of self as role model	Identify own role model, and for whom you are a role model *Participants are asked to imagine themselves as a role model for another person, and how their physical activity behavior could motivate that person to be physically active too, which helps acknowledge the positive impact of their actions.*
13		Demonstration of the behavior; Social comparison; Credible source	Watch video of Usain Bolt interview *Participants are asked to watch a video about an interview with Usain Bolt to give them a positive example of someone to has an active lifestyle, and incorporate his advice into their own physical activity behavior.*
14		Review outcome goal(s)	Reflect on PA challenge (day 9) *Participants are asked about the challenge they would set with a peer and how this resulted in higher levels of physical activity, either to motivate them to use their social support system more often or to think about ways to overcome barriers in involving social support.*
15		Review behavioral goal(s)	Reflect on goals (day 11) and make adjustments (SMART) *Participants are asked to look back at their goal of day 11, and if needed, create a new specific, measurable, achievable, relevant and time-bound goal to help them increase their step count.*
16		Focus on past success	Reflect on rewarding experience of previous physical activities *Participants are asked to think about physical activity they have performed before and its positive consequences to motivate them in engaging in physical activity to reach their goal.*
17		Reduce negative emotions; Monitoring of emotional consequences	Stress management and emotional coping *Participants are asked to watch a video with a breathing exercise that would help them in the management of stress and negative emotions, and to think how physical activity would help them in this management to motivate them in increasing physical activity levels.*
18		Self-talk	Positive labelling of upsetting experiences *Participants are asked to think about a negative experience during the intervention and their feelings, after which they are asked to relabel this situation to help them in overcoming similar situations in during future physical activity.*
19	Maintenance	Review outcome goal(s)	Reflect on barriers and coping strategies (day 7) *Participants are asked to think about the potential barrier they mentioned on day 7, and if their solution helped them in overcoming this barrier, to help them with coping strategies that might hinder reaching their physical activity goals in the future.*
20		Incompatible beliefs; Discrepancy between current behavior and goal	Imagine future self and set goals to work towards that *Participants are asked to think about themselves in the future, and to set physical activity goals for the current version of themselves to make themselves satisfied in the future.*
21		Monitoring of emotional consequences; Review outcome goal(s)	Meta-reflection of intervention (what did I learn, what did I like the most, how did I change?) *Participants are asked to reflect on the intervention and their physical activity process and to identify lessons learned that help them in engaging in physical activity in the future.*
1–21	All stages	Social reward; Feedback on behavior	Praise for effort and progress; inform participant about daily step counts *On a daily basis, participants were informed about their step count and goal progress, and positively encouraged to keep up with their physical activity or increase their physical activity levels.*

**Table 3:** Medians and interquartile range (IQR) per group of effectiveness (mean difference between average baseline step count and average step count in final week of intervention).

Variable	Effectiveness	
	N	Median (IQR^a^)
Visual & relational cues	11	1395 (1868)
Visual cues	6	1703 (1831)
Relational cues	11	785 (2460)
No cues	15	438 (4149)

a IQR = interquartile range.

## Appendix 2 Technical implementation of the benefit StepCoach app

We developed the Benefit StepCoach app as a tool to test our hypotheses. The app was developed with use of MobileCoach software (www.mobile-coach.eu) (Filler et al., 2015; Kowatsch et al., 2017), an open-source software platform for smartphone-based and chatbot-delivered behavioral interventions (eg, (Stieger et al., 2021)) and ecological momentary assessments (eg, (Tinschert et al., 2019)). The Mobile Coach platform provided the researchers with a web-based graphical user interface and allowed us to implement the needed intervention logic and content. MobileCoach uses a web server to execute the needed intervention logic and to deliver the content to the MobileCoach-based mobile applications for Apple's iOS and Android platforms. The mobile app was customized to fit the needs of this study and published in the iOS and Android app stores with the name Benefit StepCoach. One of the important features of this app was to automatically and objectively retrieve step counts of the participants. Google Fit (www.google.com/fit/) for the Android app and Apple's Health Kit (developer.apple.com/documentation/healthkit) for the iOS app were used for this purpose. Appropriate interactions were implemented, i.e. asking participants for their permission, to allow the app to access step data. Moreover, each experimental group was assigned a dedicated TCA.

## Appendix 3 Analyses with effectiveness as outcome variable

Effectiveness was measured through objective step count data retrieved from Apple Health or Google Fit (depending on the smartphone of the participant). We calculated the mean difference between the average baseline step count (measured in the week before the intervention) and the average step count in the final week of the intervention. Participants were included in the analyses if both a valid baseline step count and a minimum of 5 days step count in the final week were registered.

To test whether the intervention was effective in urging participants to increasing participants' step count (independently of the experimental condition), a (one-tailed) paired samples *t-*test was conducted. Our analyses showed a significant increase in the average step count from the baseline week (M = 3412.37, SD = 2363.17) to the final week (M = 4556.77, SD = 2545.65), t(42) = −3.975, *p* < .001, 95 % CI [−1725, −563].

In addition, due to small sample size, we conducted a nonparametric Kruskal-Wallis test to compare intervention effectiveness between the four different cue conditions. There was no significant difference in effectiveness between the conditions, *H*(3) = 2.536, *p* = .47 (see Table 3 for median and IQR per group). Also the post-hoc Mann-Whitney *U* tests comparing the three human cues conditions with the no human cues condition (*U* = 170.5, *z* = −1.007, *p* = .32), and the test comparing the condition with both visual and relational cues with the visual cues only and relational cues only groups (*U* = 91.0, *z* = −0.118, *p* = .92) showed no significant differences.
